# Malignant Melanoma of the Foot in Five Elderly Patients: Clinicopathologic Features and Treatment Outcomes

**DOI:** 10.7759/cureus.82076

**Published:** 2025-04-11

**Authors:** Jekin J Sharon, Gokulesh D G, Kamalraj M

**Affiliations:** 1 General Surgery, Madras Medical College, Chennai, IND

**Keywords:** acral melanoma, adjuvant therapy, breslow thickness, elderly patient care, foot tumors, melanoma recurrence, melanoma surgery, nodular melanoma, plantar melanoma

## Abstract

Malignant melanoma, an aggressive neoplasm of melanocytes, remains a leading cause of skin cancer-related deaths globally, with rising incidence among elderly populations. Foot melanomas are often diagnosed at advanced stages due to delayed detection and misdiagnosis. They exhibit poorer outcomes compared to melanomas at other sites. This case series presents five elderly patients (ages 58-80) with malignant melanoma of the foot, detailing their clinicopathological features, treatment strategies, and outcomes. All cases presented with advanced disease, emphasizing the challenges of late diagnosis in this demographic. Histopathological evaluation confirmed nodular, superficial spreading, and acral lentiginous subtypes, with Breslow thicknesses ranging from 1.5 mm to 3.5 cm. Management involved wide local excision, lymph node dissection, and adjuvant chemotherapy in high-risk cases. Recurrence occurred in one patient, underscoring the aggressive nature of the disease. The series highlights the necessity of early detection, multidisciplinary care, and tailored adjuvant therapies to improve outcomes in elderly patients with foot melanoma.

## Introduction

Malignant melanoma, a neoplasm of melanocytes, is recognized for its aggressive behavior and high potential for metastasis. It accounts for the majority of skin cancer-related deaths globally despite being less common than other skin cancers [1]. Over recent decades, the incidence of malignant melanoma (around 3600 cases in 2022 in India) has risen significantly, particularly among older populations, who are often diagnosed at more advanced stages of the disease [2]. This trend raises significant concern due to the typically worse prognosis linked to advanced age and the presence of comorbid conditions [3]. 

Foot and subungual melanomas are typically diagnosed with an average Breslow thickness of 5.03 mm, a critical prognostic indicator, compared to an average thickness of 0.8 mm at other cutaneous sites. Consequently, both overall and disease-free survival rates are significantly poorer for patients with foot melanomas [2]. These melanomas are more commonly observed in older adults, who often face challenges in performing self-examinations. Coupled with frequent initial misdiagnoses, these factors contribute to substantial diagnostic delays [3]. Diagnostic accuracy is further complicated by the frequent presentation of amelanotic melanomas in this location, which can mimic benign conditions such as common warts, subungual hematomas, vascular or diabetic ulcers, or other chronic inflammatory disorders prevalent in the elderly population [4].

This case series presents five elderly patients diagnosed with malignant melanoma of the foot, focusing on their clinical presentation, diagnostic findings, therapeutic interventions, and outcomes.

## Case presentation

Case 1 

A 65-year-old male presented with a three-month history of a progressively enlarging, painful swelling on the lateral aspect of his left foot with restricted mobility. There was no history of trauma or other swellings. Clinical examination revealed a firm, irregularly bordered, fixed 5 x 4 cm mass, raising suspicion of malignancy. An incision biopsy confirmed nodular-type malignant melanoma. Staging with CT of the chest and brain showed no distant metastasis, while CT of the lower limb and pelvis revealed enlarged lymph nodes suggestive of locoregional metastatic spread. MRI of the lower limb was performed to delineate the tumor from surrounding soft tissues and assess the depth of invasion.

**Figure 1:**
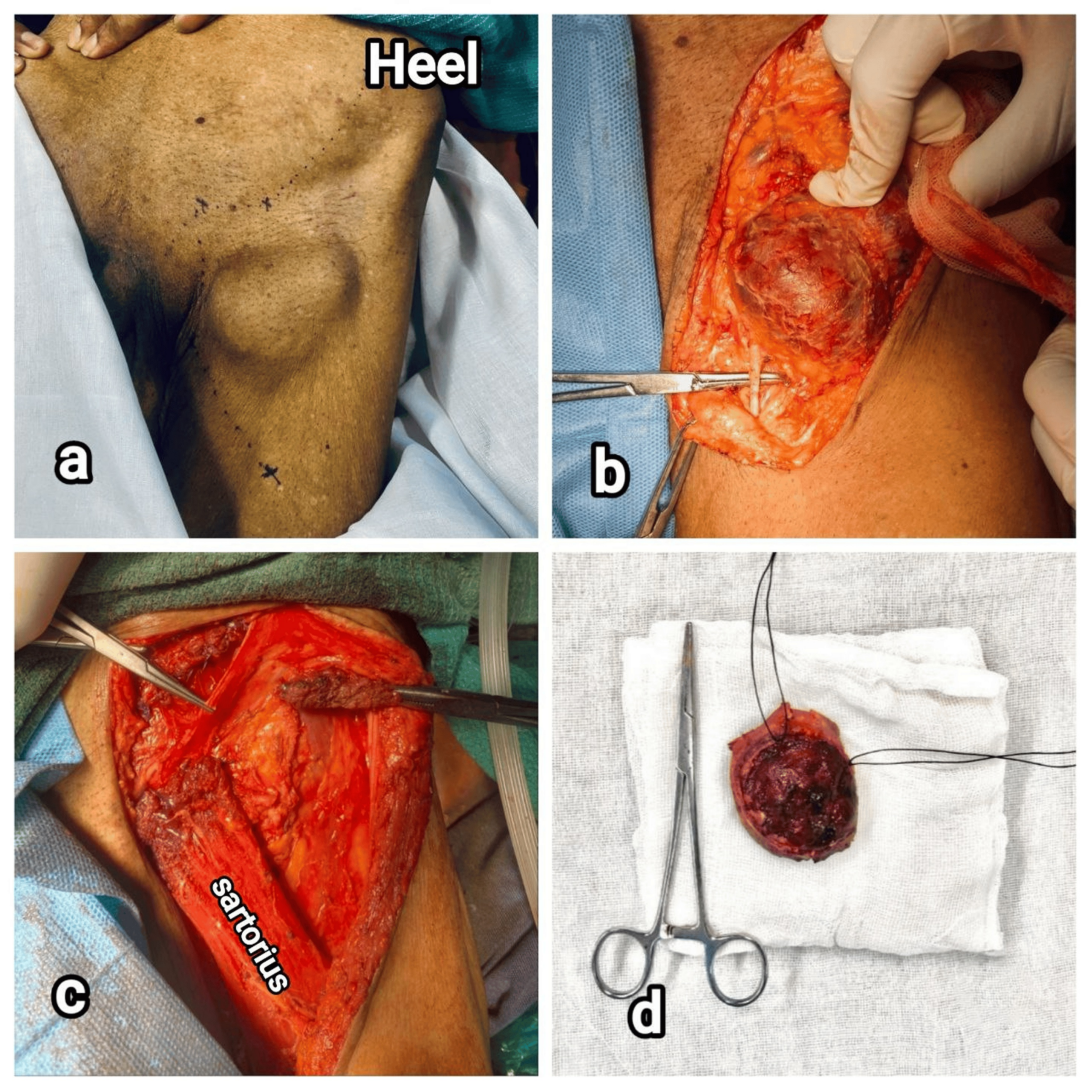
Intraoperative images of patient 1. (a) Nodular melanoma in the dorsolateral aspect of left foot, (b) tumor near the superficial peroneal nerve (indicated by artery forceps) after dissection, (c) left-side ilioinguinal dissection showing femoral triangle, and (d) tumor specimen removed

The case was reviewed by a multidisciplinary tumor board, which recommended a comprehensive treatment approach. Wide local excision of the tumor was performed, along with ilioinguinal lymph node dissection on the left (Figure 1). Histopathological analysis revealed a well-differentiated nodular melanoma measuring 5 x 4.6 x 1.4 cm and involving the deep dermis. The maximum tumor (Breslow) thickness was 1.4 cm, and the anatomic (Clark) level was IV. The tumor exhibited lymphovascular invasion, a mitotic rate of 1-2 per high-power field (hpf), and focal tumor-infiltrating lymphocytes at the base. Tumor regression and neurotropism were absent. Metastatic deposits were identified in all six dissected iliac and inguinal lymph nodes, with the largest measuring 9 x 6 cm. The tumor was staged as pT4bN3bMx. All surgical margins (ranging from 0.5 to 1.8 cm) were tumor-free.

The postoperative recovery was uneventful, with complete wound healing. Given the advanced stage and high-risk features, adjuvant chemotherapy was initiated to reduce the risk of recurrence as per the advice of our institutional tumor board. Three cycles of dacarbazine with cisplatin and Adriamycin were administered, with each cycle lasting 21 days. At the six-month follow-up, there was no evidence of recurrence or metastasis, and the patient remains under follow-up.

Case 2

A 75-year-old male presented with a pigmented, ulcerated lesion over the plantar aspect of his right foot, persisting for one year. He reported itching and pain associated with the lesion but had no significant systemic symptoms such as fever or weight loss. The clinical diagnosis of malignant melanoma of the right foot was confirmed with an edge wedge biopsy. Histopathological examination revealed hyperplastic stratified squamous epithelium infiltrated by malignant cells arranged in sheets and clusters. The tumor extended into the papillary dermis. The intervening stroma showed intracytoplasmic melanin pigments and lymphohistiocytic infiltrates.

Contrast-enhanced computed tomography of the abdomen and pelvis revealed multiple enlarged, homogeneously enhancing inguinal lymph nodes bilaterally. The largest node on the right measured 1.4 x 1.1 cm, while the largest node on the left was 1.3 x 0.9 cm. There was no evidence of pre- or para-aortic nodal enlargement. Other abdominal organs appeared normal without evidence of metastatic spread. An ultrasound-guided fine-needle aspiration cytology was suggestive of reactive lymphadenitis. Biochemical tests of liver function were within normal limits. Given the diagnosis of malignant melanoma with probable lymphatic spread, a comprehensive treatment plan was formulated.

**Figure 2:**
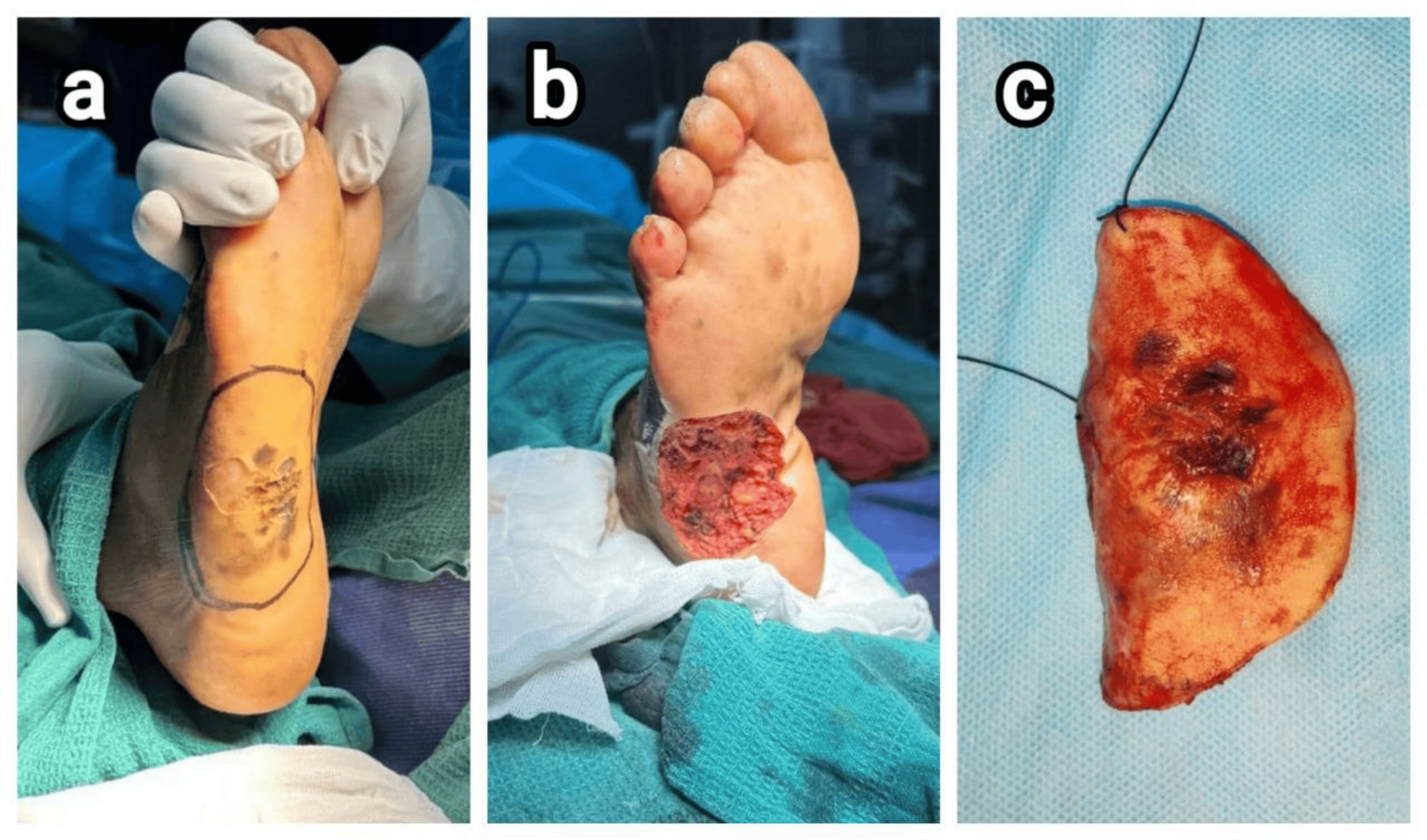
Intraoperative images of patient 2. (a) Plantar lesion in the lateral border of the right foot and 2-cm margin marked all around, (b) after wide local excision, and (c) excised specimen

The surgical procedure included a wide local excision of the lesion with 2 cm margins all around and ilioinguinal dissection under spinal anesthesia (Figure 2). Meticulous postoperative wound care and pain management were provided. The histopathology report confirmed malignant melanoma, and all surgical margins were free from tumor infiltration. There were no satellite nodules, lymphovascular invasion, neurotropism, or tumor regression. The mitotic rate was 2-3 per hpf. A Breslow thickness of 1.5 mm was observed, and Clarke's anatomical stage II was designated. The inguinal lymph node specimen was reported to be suggestive of reactive lymphadenitis. The patient remained recurrence-free at the six-month follow-up.

Case 3

A 63-year-old female presented with a blackish swelling on her right foot, which she had initially noticed a year prior. The lesion had progressively increased in size and became associated with pain over the last six months. Clinical examination revealed a swelling over the heel of the right foot with ulceration and blackish discoloration. The swelling measured 6 x 6 cm, had irregular borders, and was fixed to the underlying muscles. Associated right-sided inguinal lymphadenopathy was also noted. A clinical diagnosis of malignant melanoma was made. A PET scan showed a hypermetabolic lesion in the right foot, consistent with the diagnosis of malignant melanoma, as well as enlarged hypermetabolic inguinal lymph nodes.

**Figure 3:**
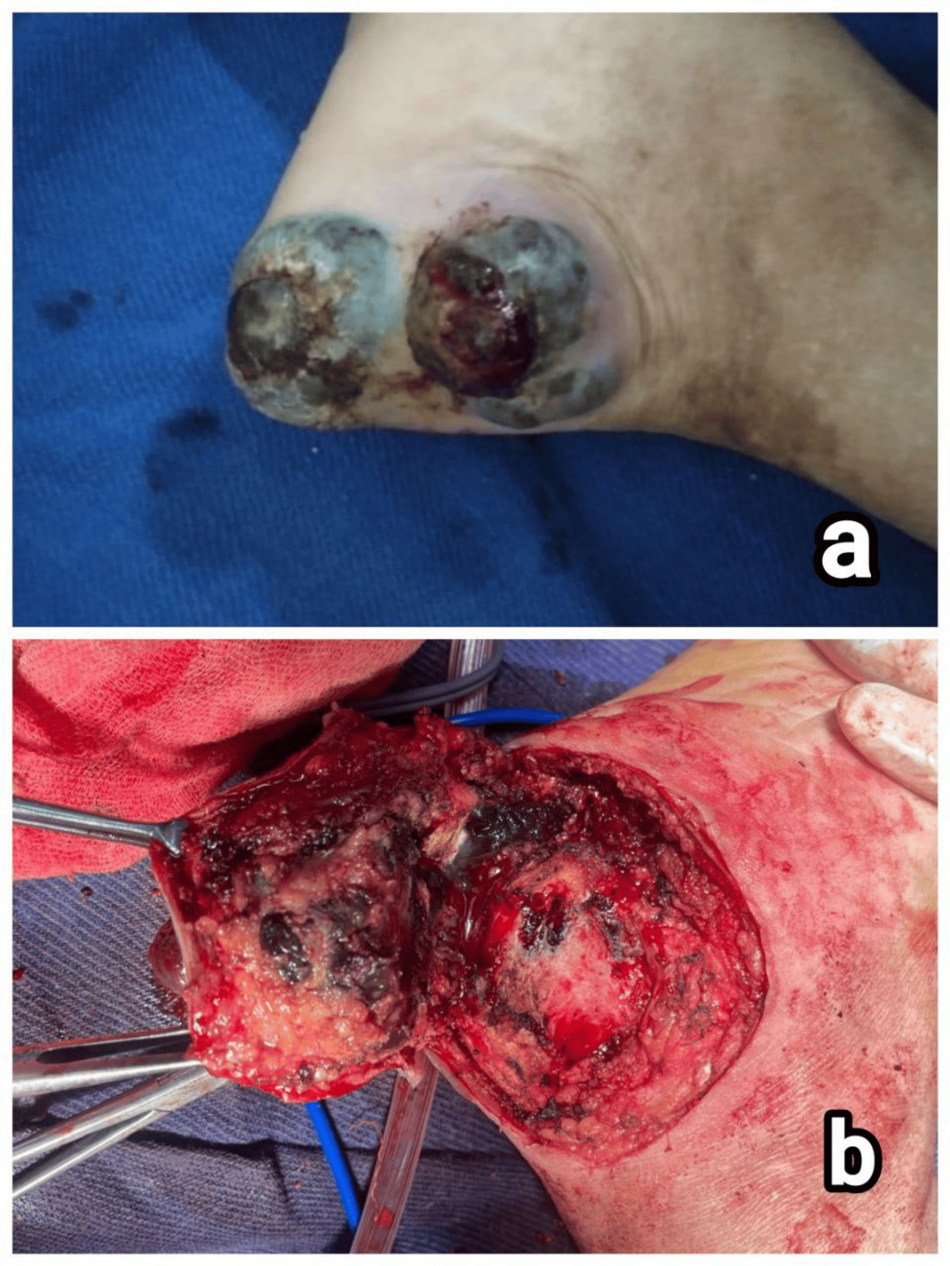
Intraoperative images of patient 3. (a) Blackish pigmented, ulcerated, nodular lesion in the heel of right foot and (b) wide local excision of tumor

The patient underwent a wide local excision of the tumor with inguinal lymph node dissection under spinal anesthesia. A 2-cm margin was taken around the tumor, and it was excised completely (Figure 3). The specimen was sent for histopathological examination. The excised tissue included a skin-attached soft tissue fragment measuring 7.5 x 4.5 x 5 cm, with a tumor depth of 3.5 cm. The tumor was nodular and brown in appearance. The inguinal lymph node dissection revealed 11 nodes, with the largest measuring 3.2 x 5.1 cm. 

Histopathological examination confirmed a diagnosis of malignant melanoma, not otherwise specified, with a Breslow thickness of 3.5 cm. The tumor exhibited ulceration, a mitotic rate of 5-10 per hpf, Clark level V invasion, and lymphovascular involvement. The deep margin showed tumor infiltration, while other margins were free of tumor involvement. Eight out of nine examined lymph nodes were involved. The pathological staging was determined as pT4bN3bMx. The postoperative period was uneventful. Adjuvant therapy based on dacarbazine, cisplatin, and Adriamycin was initiated, and regular follow-up was conducted to monitor wound healing and watch for any signs of recurrence. At the six-month follow-up, there was no evidence of recurrence.

Case 4

**Figure 4:**
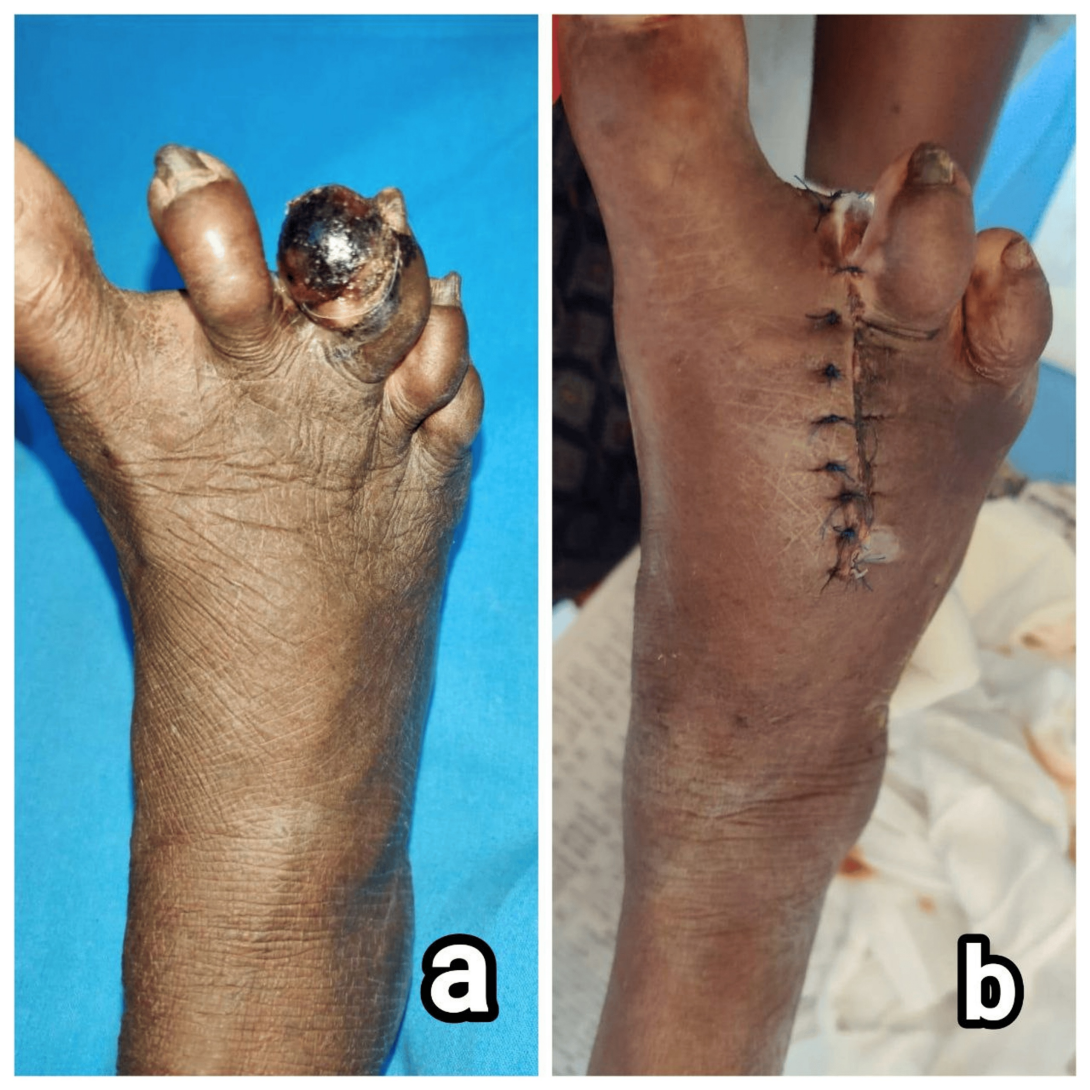
Clinical images of patient 4. (a) Blackish pigmented, ulcerated, nodular lesion in the third digit of the right foot and (b) delayed primary wound closure after Ray’s amputation of the second and third digits

An 80-year-old female presented with a six-month history of swelling and blackish discoloration affecting the medial aspect of the right third toe. The patient denied any history of involuntary movements, limb weakness, unsteadiness, or changes in voice. Physical examination revealed stable vital signs, with no abnormalities detected in the cardiovascular, respiratory, abdominal, or central nervous systems. Local examination of the right foot demonstrated a blackish, nodular swelling measuring 1.5 × 0.8 × 0.2 cm on the medial aspect of the third toe. The discoloration extended across the entire medial side of the toe and into the second interdigital space (Figure 4a). Based on clinical findings, a provisional diagnosis of malignant melanoma was made.

Laboratory investigations revealed elevated bilirubin and serum glutamic-oxaloacetic transaminase (SGOT) levels, raising suspicion of hepatic involvement. A PET scan identified a metabolically active nodular skin lesion on the medial aspect of the right third toe, consistent with a primary malignancy. No significant regional lymphadenopathy, pulmonary nodules, or liver lesions were observed. PET scan showed a metabolically active nodular skin lesion without significant regional lymphadenopathy/pulmonary nodules/other lesions elsewhere in the body. Histopathological examination of an incisional biopsy specimen revealed hyperkeratotic stratified squamous epithelium with ulceration and infiltrating neoplastic cells arranged in nests and cords. Immunohistochemical analysis demonstrated diffuse strong membranous positivity for HMB-45 in over 90% of tumor cells, confirming the diagnosis of malignant melanoma. Tumor invasion extended to the deep dermis.

The final diagnosis of malignant melanoma of the right foot with moderate dysplasia and no evidence of regional metastasis was made. Surgical management involved Ray’s amputation of the second and third digits of the right foot. Histopathology of the resected specimen revealed an acral lentiginous melanoma of size 1.5 x 0.8 x 0.2 cm with a Breslow thickness of 4 mm. Clark anatomical stage III with a mitosis rate of 1-2 per hpf was observed. There was no lymphovascular invasion, neurotropism, or macrosatellite nodules. The postoperative course was uneventful, with adequate pain control and wound care facilitating optimal healing (Figure 4b). Delayed primary closure of the surgical wound was performed after one month. Adjuvant therapy was not administered. At the six-month follow-up, the patient remained recurrence-free.

Case 5

A 58-year-old female was admitted with an ulcerated lesion over the plantar aspect of her right foot, accompanied by pain and discomfort for the past 16 months. On examination, a swelling of 8 x 5 x 5 cm was present on the lateral aspect of the right heel with a blackish discoloration. It was firm in consistency and fixed to the underlying muscles and fascia. Clinical diagnosis was malignant melanoma of the right foot with suspected inguinal lymph node involvement. Incision biopsy also confirmed the diagnosis. CT scans of the abdomen, chest, and brain were normal, while CT pelvis showed enlarged inguinal lymph nodes.

**Figure 5:**
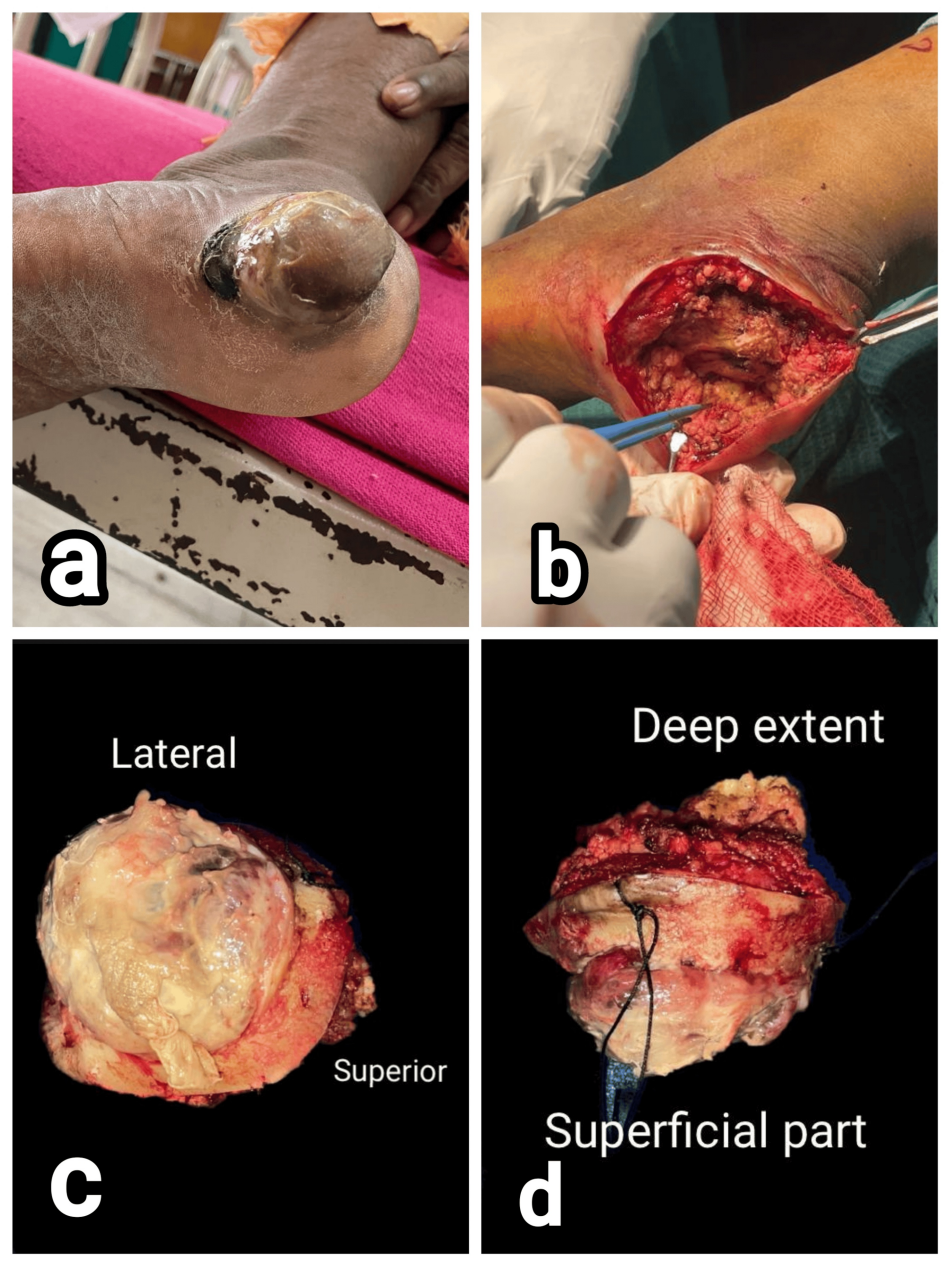
Clinical images of patient 5. (a) Non-pigmented, nodular lesion in the heel of left foot, (b) wide local excision of the tumor, and (c and d) specimen sent for histopathology

Management included a wide local excision of the tumor with inguinal lymph node dissection (Figure 5). The specimen was sent for histopathological examination. Post-operatively, the patient was given intravenous antibiotics to prevent infection, analgesics for pain management, and regular monitoring of vital signs. Daily dressing changes and monitoring for signs of infection were part of the wound care routine.

Histopathological examination revealed a skin-attached soft tissue fragment measuring 6 x 6 x 5 cm, with the tumor measuring 7.5 x 4.5 x 5 cm and extending 3.5 cm from the adjacent skin. Microscopic examination identified the tumor as a nodular melanoma with a brown nodular appearance, the presence of lymphovascular invasion, and the absence of neurotropism. Tumor infiltrating lymphocytes were present but not brisk, and out of the nine lymph nodes examined, eight were involved. The margins were clear from tumor infiltration except for the deep margin, which was formed by the tumor. Pathological staging was determined as pT4bN3bM1, with a maximum Breslow thickness of 3.5 cm, a mitotic rate of 5-10 per hpf, and an anatomic (Clark) level of V. All surgical margins were clear of tumor infiltration, except for the deep margin, which was formed by the tumor. Since the patient was not willing for re-excision or radiotherapy, adjuvant chemotherapy with dacarbazine, cisplatin, and Adriamycin was started.

**Figure 6:**
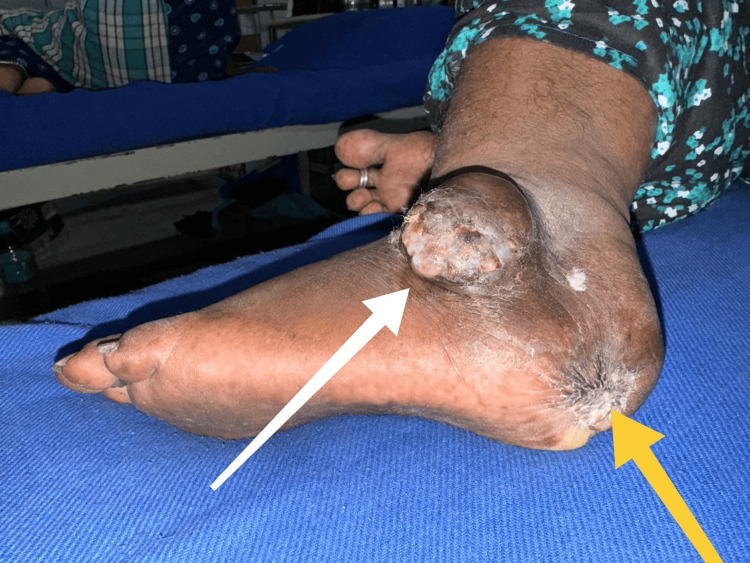
Clinical image of patient 5 showing the recurrent lesion in the lateral aspect of left foot (white arrow) and healed scar at the site of primary lesion (yellow arrow)

**Figure 7:**
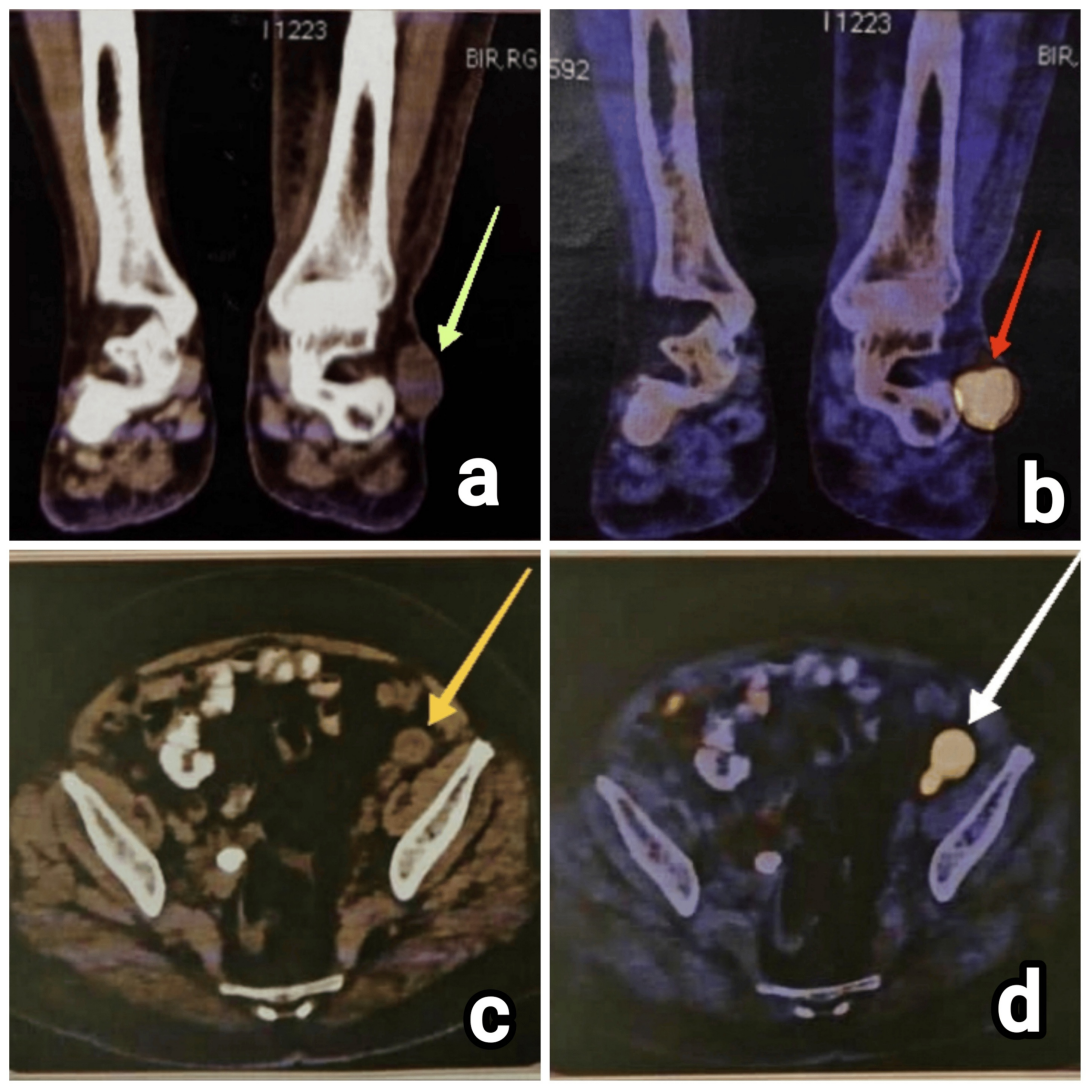
Imaging investigations of patient 5. (a) CT image of the left leg with ankle (coronal section) showing the recurrent lesion in the lateral aspect (green arrow), (b) PET scan image showing the corresponding increased metabolic uptake in the recurrent lesion in the lateral aspect of the left foot (red arrow), (c) left iliac lymph node as visualized in CT scan (yellow arrow), and (d) PET scan showing increased metabolic uptake in the corresponding left iliac lymph node (white arrow) CT, computed tomography; PET, positron emission tomography

Regular follow-up visits were scheduled to monitor for signs of recurrence or metastasis and for wound care. There was a recurrence at the six-month follow-up. PET scan identified metabolically active left popliteal and external iliac lymph nodes apart from a subcentimetric subcutaneous soft tissue density nodule in the anteromedial aspect of the middle third of the left leg (Figures 6, 7). Amputation was recommended; however, the patient declined the procedure. Hence, a wide local excision was performed, and paclitaxel-carboplatin adjuvant chemotherapy was initiated. A summary of all five cases is given in Table 1.

**Table 1:** Clinicopathological data of five elderly patients with melanoma of the foot

Characteristics	Patient 1	Patient 2	Patient 3	Patient 4	Patient 5
Age, sex	65, male	75, male	63, female	80, female	58, female
Site and laterality	Left foot, dorsolateral	Right foot, lateral plantar	Right foot, heel	Right foot, third digit	Right foot, heel
Type	Nodular, non-pigmented	Ulcerative, pigmented	Nodular, pigmented	Nodular, pigmented	Nodular, non-pigmented
Duration of illness	3 months	12 months	14 months	6 months	16 months
Size of lesion (cm)	5x4x1.5	4x2.5	7.5x5x4.5	1.5x0.8x0.2	6.5x6x5.5
Lymph node enlargement	Present	Present	Present	Absent	Present
Breslow thickness	1.4 cm	1.5 mm	3.5 cm	4 mm	11 mm
Clark anatomic level	IV	II	V	III	V
Ulceration	Absent	Present	Present	Present	Present
Macrosatellite nodules	Absent	Absent	Absent	Absent	Absent
Mitosis rate	1-2/hpf	2-3/hpf	5-10/hpf	1-2/hpf	5-10/hpf
Lymphovascular invasion	Present	Absent	Present	Absent	Absent
Neurotropism	Absent	Absent	Absent	Absent	Absent
Tumor infiltrating lymphocytes	Present at base	Present at base	Present, not brisk	Present, not brisk	Absent
Tumor regression	Absent	Absent	Absent	Absent	Absent
Resected margins	All margins free from tumor infiltration	All margins free from tumor infiltration	Tumor infiltration at deep margin alone	All margins free from tumor infiltration	All margins free from tumor infiltration
Histological type	Nodular	Superficial spreading	Malignant melanoma not otherwise classified	Acral lentiginous	Malignant melanoma not otherwise classified
TNM stage classification	pT4bN3bMx	pT2bN0M0	pT4bN3bMx	pT3bN0Mx	pT4bNxMx
Immunohistochemistry	S100+ HMB+	S100+ HMB+	S100+ HMB+	S100+ HMB+	S100+ HMB-
Treatment modality	Surgery + adjuvant chemotherapy	Only surgery	Surgery + adjuvant chemotherapy	Only surgery	Surgery + adjuvant chemotherapy
Recurrence at 6 months	No	No	No	No	Yes

## Discussion

This case series highlights the complexities and challenges inherent in managing malignant melanoma, particularly in elderly patients. Each case underscores critical aspects of diagnosis, treatment, and follow-up, reflecting current clinical practices and the evolving understanding of this aggressive disease. 

Malignant melanoma is notorious for its aggressive behavior and high metastatic potential. In elderly patients, diagnosis is often delayed, leading to more advanced disease at presentation, which significantly impacts prognosis and treatment outcomes [1]. In this series, all patients presented with advanced disease, emphasizing the need for heightened clinical vigilance and early intervention in this vulnerable population. Histopathological examination remains the cornerstone of diagnosis, providing essential details such as tumor thickness, ulceration status, mitotic rate, and the presence of lymphovascular invasion--all of which are critical for accurate staging and prognostication [4]. For instance, in Case 1, histopathological findings of nodular melanoma with significant lymphovascular invasion and lymph node involvement necessitated aggressive surgical intervention and consideration of adjuvant therapies. 

Surgical excision with clear margins is the primary treatment for localized malignant melanoma. However, the extent of surgery, including lymph node dissection, depends on the disease stage and the presence of metastasis [5]. In Case 2, wide local excision and ilioinguinal dissection were performed, aligning with standard procedures aimed at achieving local control and reducing the risk of regional spread. Given the high risk of recurrence and metastasis in advanced melanoma, adjuvant therapies such as immunotherapy and targeted therapy are increasingly recommended after surgery [6]. Cases 1, 3, and 5 illustrate the importance of a multidisciplinary approach, incorporating oncological assessments to evaluate the suitability of adjuvant treatments. Immunotherapy, in particular, has shown promise in improving survival outcomes in patients with high-risk melanoma [7]. 

Managing melanoma in elderly patients presents unique challenges, as these individuals often have comorbidities that can complicate treatment plans and influence overall prognosis. For example, in Case 5, the patient’s elevated bilirubin and SGOT levels suggested possible liver involvement, necessitating careful consideration in her treatment strategy. Regular follow-up is crucial for the early detection of recurrence or metastasis. Surveillance strategies typically include physical examinations, imaging studies, and blood tests [8]. In this series, all patients were scheduled for regular follow-up visits to monitor their postoperative status and address any emerging complications. Patient education plays a vital role in recognizing symptoms of recurrence and ensuring adherence to follow-up schedules. 

The management of malignant melanoma demands a multidisciplinary approach, integrating surgical excision, adjuvant therapies, and close follow-up to monitor for recurrence and metastasis [4]. Early detection and accurate staging are pivotal in determining the appropriate treatment strategy and improving patient outcomes [5]. Histopathological examination remains the gold standard for diagnosis, offering critical insights into tumor type, depth, and the presence of lymphovascular invasion, which are indispensable for staging and prognostication [6]. 

Melanoma of the foot is notorious, as it is associated with poor survival outcomes (68.4%; five-year survival) compared to melanoma of other sites (91.7%). The disease also presents at an advanced stage due to multiple factors such as absence of pain and proper visualization of the lesion. Most plantar melanomas also harbor detrimental loss of BRAF and N-RAS mutations. Adams et al. have also reported a higher rate of sentinel lymph node positivity for plantar melanomas (51.6%) [9].

The management and outcomes of malignant melanoma in elderly patients have been extensively explored in the literature, providing valuable insights into clinical challenges and therapeutic strategies. A retrospective analysis by Hodi et al. of 20 elderly patients (aged 70 and above) with advanced melanoma demonstrated that aggressive surgical intervention combined with adjuvant therapy significantly improved overall survival. The study emphasized the importance of early detection and regular follow-up to monitor for recurrence. Patients who received combination therapy (surgery plus adjuvant immunotherapy) had better outcomes compared to those who underwent surgery alone [10]. A case report by Schadendorf et al. described a 78-year-old female with malignant melanoma on the lower limb, presenting similarly to the cases in our series. The patient underwent wide local excision and sentinel lymph node biopsy, followed by adjuvant nivolumab therapy. Histopathological examination revealed nodular melanoma with a Breslow thickness of 2.8 cm and lymphovascular invasion. The patient remained disease-free for 18 months after treatment, underscoring the efficacy of combined surgical and immunotherapy approaches in managing advanced melanoma in elderly patients [11]. 

Another case series by Faries et al. evaluated the surgical outcomes of 10 elderly patients with melanoma who underwent wide local excision and lymph node dissection. The findings indicated that while surgical resection with clear margins was achievable, the risk of postoperative complications was higher in elderly patients due to comorbidities. The study advocated for a multidisciplinary approach to optimize perioperative care and enhance recovery [12]. A longitudinal study by Garbe et al. on elderly melanoma patients (aged 75 and above) reported that early-stage diagnosis and prompt surgical intervention were crucial for long-term survival. The study highlighted that patients who received adjuvant therapy, particularly immune checkpoint inhibitors, had improved disease-free survival rates. Regular monitoring and follow-up were essential to detect and manage recurrences effectively [13]. Larkin et al. discussed the management of recurrent melanoma in an 82-year-old male, highlighting the challenges of treating elderly patients with recurrent disease. The patient underwent a second surgical excision followed by pembrolizumab therapy. Despite the advanced stage and recurrence, the patient achieved partial remission, demonstrating the potential benefits of novel immunotherapeutic agents in recurrent melanoma cases [14]. A comparative study by Eggermont et al. analyzed the outcomes of elderly melanoma patients treated with surgery alone versus those who received adjuvant therapy. The study concluded that adjuvant therapy significantly reduced the risk of metastasis and recurrence, particularly in patients with lymphovascular invasion and high mitotic rates. The findings supported the integration of adjuvant therapies into the standard treatment protocol for high-risk elderly melanoma patients [15]. 

The findings from this case series underscore the importance of individualized care plans that account for the patient’s overall health status, comorbidities, and preferences. Early detection through vigilant screening and prompt intervention remains key to improving outcomes in elderly melanoma patients [2]. Moreover, advancements in adjuvant therapies offer hope for better management of high-risk cases, though further research is needed to optimize these treatments for elderly populations.

## Conclusions

This case series underscores the complexities and challenges associated with managing malignant melanoma of the foot in elderly patients, which is often diagnosed at advanced stages of the disease. The study highlights the critical importance of early detection, accurate histopathological diagnosis, and precise staging to guide treatment strategies. Surgical resection with clear margins remains the cornerstone of treatment, while adjuvant therapies, such as chemotherapy, play a vital role in reducing recurrence risk, particularly in high-risk cases. The findings from this series align with existing literature, reinforcing the value of a multidisciplinary approach that integrates surgical intervention, adjuvant therapies, and vigilant follow-up.
